# Impact of Premature Ovarian Failure on Mortality and Morbidity among Chinese Women

**DOI:** 10.1371/journal.pone.0089597

**Published:** 2014-03-06

**Authors:** Xiaoyan Wu, Hui Cai, Asha Kallianpur, Honglan Li, Gong Yang, Jing Gao, Yong-Bing Xiang, Bu-Tian Ji, Yu-Tang, Wei Zheng, Xiao-Ou Shu

**Affiliations:** 1 Division of Epidemiology, Department of Medicine, Vanderbilt University School of Medicine and Vanderbilt-Ingram Cancer Center, Nashville, Tennessee, United States of America; 2 Currently at Department of Biostatistics, Public Health College, Harbin Medical University, Harbin, P. R. China; 3 Division of General Internal Medicine and Public Health, Department of Medicine, Vanderbilt University School of Medicine, Nashville, Tennessee, United States of America; 4 Department of Epidemiology, Shanghai Cancer Institute, Shanghai, P. R. China; 5 Occupational Epidemiology Branch, National Cancer Institute, National Institutes of Health, Bethesda, Maryland, United States of America; VU University Medical Center, Netherlands

## Abstract

**Objective:**

To evaluate associations of premature ovarian failure (POF) with mortality and morbidity in Asian populations.

**Methods:**

We identified 1,003 cases of POF among 36,402 postmenopausal women who participated in the Shanghai Women's Health Study, a population-based cohort study. Cox regression and logistic regression models were applied in data analysis.

**Results:**

After adjustment for potential confounding factors, we found that POF increased the risk of total and cancer-specific mortality (HR (95%CIs): 1.29 (1.08–1.54) and 1.38 (1.05–1.81), respectively). POF was also associated with high prevalence of autoimmune disease (OR (95%CI): 1.56 (1.04–2.35)) but decreased incidence of breast cancer (OR (95%CI): 0.59 (0.38–0.91)). Similar results were observed when hormone replacement therapy users were excluded from the analysis. POF is associated with high waist-to-hip ratio.

**Conclusions:**

Our results suggest that women with POF experience increased mortality and that these women may benefit from heightened surveillance and appropriate interventions.

## Introduction

Premature ovarian failure (POF) (also known as premature menopause) is defined as the cessation of menses associated with secondary amenorrhea, sex steroid deficiency, and elevated serum levels of gonadotropins before the age of 40 [1], [2]. While POF can occur spontaneously, it may be induced by surgery for gynecological disorders, systemic chemotherapy or radiotherapy, or pelvic irradiation. Many potential etiologies for POF have been suggested [3], but the majority of spontaneous cases are idiopathic. POF affects an estimated 1% of women under the age of 40 [4]. As expected, the condition is more common among cancer survivors [1]. Previous studies have shown that women who experience POF have increased overall mortality and are at increased risk of cardiovascular disease (CVD), neurocognitive disorders such as Parkinson's disease, endocrine and autoimmune disorders, as well as subfertility [5]–[14]. All of these studies focused on the specific health consequences of POF in Western populations. A cross-sectional survey of women aged 40–55 years, conducted in the US, showed that the prevalence of POF varied by ethnicity, ranging from 1.4% among women of African-American and Hispanic ancestry to 1.0% among European-ancestry women, 0.5% among Chinese-ancestry women, and 0.1% among Japanese-ancestry women. However, the numbers of women of Asian ancestry in that study, particularly women with POF, were very small [15], and to date, little is known about POF or its impact on morbidity and mortality among Asian women.

The objective of the present study was to evaluate the prevalence of POF and influence of POF on overall mortality and cancer-and CVD-related morbidity and mortality among Chinese women who participated in a large, prospective cohort study conducted in Shanghai, China, the Shanghai Women's Health Study (SWHS).

## Materials and Methods

### Ethics statement

The study protocols were approved by the Institutional Review Boards of Vanderbilt University, Nashville, Tennessee and the Shanghai Cancer Institute, Shanghai, China. Written, informed consent was obtained from all participants.

### Study population

Between 1996 and 2000, 74,941 women aged 40–70 years from 7 urban districts of Shanghai were enrolled in the SWHS, an ongoing, population-based, prospective cohort study with a high participation rate (92%) [16]. At study enrollment, a detailed in-person interview was administered by trained personnel to all study participants using a structured questionnaire to gather information on demographics, dietary intake, physical activity, occupational history, personal habits and lifestyle, past medical and surgical history, family cancer history, menstrual and reproductive history, and hormone use. Anthropometric measurements, including height, weight, waist and hip circumferences, were also taken during the baseline survey. Four biennial in-person follow-ups for all living cohort members have been conducted between 2000 and 2002, 2002 and 2004, 2004 to 2006, and 2008 to 2010 with response rates of 99.8%, 98.7%, 95.2%, and 92%, respectively.

A total of 37,168 (46.8%) women were postmenopausal at study enrollment. We excluded women who were lost to follow-up after study enrollment (*N* = 6), women who reported cessation of menstruation before age 40 due to hysterectomy alone and women who had undergone hysterectomy with unilateral oophorectomy (*N* = 743), and women with missing BMI or WHR information (*N* = 17). Data from 36,402 postmenopausal women were included in the final analytic dataset.

### Ascertainment of POF

We used the World Health Organization's definition of menopause, *i.e.*, the absence of menstruation for ≥12 months [17], in our study. At baseline, participants were asked whether they had menstrual periods, the date of the last menstrual period, and for women who reported cessation of menses, whether cessation of menses was spontaneous, induced by hysterectomy or oophorectomy, or due to another cause. Menopause before age 40 was defined as POF according the previously published definition [18] . POF was further classified as spontaneous, iatrogenic, other-cause, or idiopathic, according to the reasons given for cessation of menses in our study. A total of 1,003 women met this definition of POF, including 733 spontaneous cases, 167 iatrogenic cases, 102 cases due to other causes, and one case of idiopathic POF.

### Outcome ascertainment

The cohort was followed by biennial in-person follow-up surveys and record linkage with the Shanghai Cancer Registry and the Shanghai Vital Statistics Registry which provides near 100% coverage of cancer incidence and mortality of our study participants. Subjects were censored at December 31, 2009 for mortality and cancer outcomes. Information about cause of death was collected from death certificates and coded according to the International Classification of Disease, 9th Revision (ICD-9) [19]. The cause-specific deaths we examined included deaths due to CVD (ICD-9 codes: 390–459) and cancer (ICD-9 codes: 140–208). Incident cancer cases, including breast, ovarian, and uterine cancer, were verified whenever possible by reviewing medical charts from the diagnostic hospital. Prevalence of chronic diseases, including CVD (including coronary heart disease [CHD] and stroke), diagnosis of hypertension, hyperlipidemia, diabetes mellitus, autoimmune disease (including systemic lupus erythematosus and rheumatoid arthritis), ovarian cysts, fatty liver disease, and bone fractures, was ascertained by combining baseline and follow-up survey data. Information about the above diseases was collected by asking questions like: “Have you ever been diagnosed with this disease,” followed by, “What was the date of diagnosis, and at which hospital were you diagnosed?”

### Other demographic, lifestyle, and clinical variables

In the analysis, we used information on socio-demographic factors collected at baseline, including age at study enrollment (years), occupation (professional, clerical, manual laborers, housewife/retired), family income in yuan/year (<10,000, 10,000–19,999, 20,000–29,999, >30,000), current smoking (yes/no), age at menarche (years), nulliparity (yes/no), use of hormone replacement therapy (HRT) (yes/no), and family history of ovarian cancer (yes/no) and breast cancer (yes/no). Body mass index (BMI, kg/m^2^) was calculated as weight (kg) divided by the square of height in meters (m^2^), and waist-to-hip ratio (WHR) was calculated as waist circumference (cm) divided by hip circumference (cm). Data on daily physical activity at baseline was collected using a validated questionnaire for this purpose, with physical activity measured in metabolic equivalents (MET-h/day/year) [20]. Energy intake was estimated based on data from a validated food-frequency questionnaire, as previously described [21]. Because study participants were recruited to the study at age 40 or older, all the above mentioned information was collected after POF.

### Statistical analysis

In case-control comparisons of selected demographic and other factors with adjustment for age at study enrollment, analysis of variance (ANOVA) was used for continuous variables and logistic regression was conducted for categorical variables. Length of follow-up for each participant was calculated from the interval between the baseline survey and death or last follow-up. Hazard ratios (HRs) and their 95% confidence intervals (95% CIs) were calculated to assess the effect of POF on all-cause, CVD-, or cancer-specific mortality and cancer incidence using Cox proportional hazards models. We also conducted analyses excluding HRT users. To evaluate the association of anthropometric measurements and POF, participants were categorized by quartiles of BMI, height, weight, WHR, waist and hip circumferences, and the lowest quartile was used as the reference category. Tests for trend across categories of anthropometric measurements were performed using median values in each quartile as continuous variables. Odds ratios (ORs) and their 95% CIs were estimated to measure the association between anthropometric measurements and POF, and between POF and morbidity using logistic regression models. Multivariate-adjusted regression models included the following potential confounders: age at study enrollment (years), family income (4 categories), occupation (4 categories), BMI (kg/m^2^), WHR (continuous), current smoking (yes/no), nulliparity (yes/no), age at menarche (years), type of menopause (4 categories), and HRT use (yes/no). BMI and WHR were not included in models used to calculate ORs for the association of POF with anthropometric measurements. Multivariable analyses were stratified by birth calendar year for Cox proportional regression models. The variance inflation factor for the various anthropometric variables was 1.16 or less, suggesting that no multicollinearity exists among these variables.

All analyses were performed using SAS (version 9.2, SAS Institute, Inc., Cary, NC) and a two-sided *P*-value<0.05 was considered statistically significant.

## Results

Among the 36,402 postmenopausal women included in the analyses, 1,003 (2.8%) met the criteria for POF, 93 of whom reported to have used HRT at baseline. Characteristics of women with POF (cases) and without POF (non-cases) at baseline are presented in Table 1. Compared with non-cases in our study, women with POF were significantly younger and less likely to have a professional occupation and were more likely to have low family income, to be older on reported age at menarche, current smokers, HRT users, or nulliparous. Menopause due to surgery or other iatrogenic causes was less prevalent among POF cases than non-cases. Differences in measured BMI, regular exercise participation, and family history of ovarian or breast cancer between these two groups were not statistically significant. Associations of BMI and WHR with POF are presented in Table 2. POF was significantly more prevalent among women with a high WHR (multivariate-adjusted OR (95%CI): 1.26 (1.04–1.51)) for the highest *vs.* lowest quartiles of WHR (*P*-trend = 0.005)), but no significant association between BMI and POF was observed in this study. Interestingly, POF was also slightly more prevalent in women of lower weight (*P*-trend = 0.021); however, height and hip circumference were not associated with the risk of POF (data not shown).

**Table 1:** Comparison of selected characteristics at baseline between POF cases and non-cases in the Shanghai Women's Health Study.

Characteristics	POF Cases (*N* = 1,003)	Non-cases (*N* = 35,399)	*P* value^a^
Age at recruitment (years), mean (SD)	58.79(9.00)	60.18(6.46)	<0.001
Age at recruitment (years, %)			
<55.08	24.56	32.40	
55.08-	25.44	14.56	
61.25-	24.96	22.43	
65.58-	25.04	30.61	<0.001
Follow-up years (median (q1, q3))	11.15(10.29,11.82)	11.23(10.35,11.85)	0.0216**
Years between menopause and last follow-up	34.42(25.13,39.38)	21.71(16.21,27.21)	<0.001**
Years between menopause and baseline survey	24.02(14.19,28.68)	11.00(5.08,16.37)	<0.001**
Body-mass index, mean (SD)	24.48(3.75)	24.65(3.61)	0.492
Waist-to-hip ratio, mean (SD)	0.83(0.06)	0.82(0.06)	0.003
Occupation, professional (%)	21.54	30.25	<0.001
Family income, <10,000 yuan/year (%)	21.73	19.62	0.002
Regular exercisers (%)	45.96	49.04	0.368
Current smokers (%)	5.98	3.34	<0.001
Age at menarche (years), mean (SD)	15.20(2.04)	15.15(1.81)	0.032
Nulliparity (%)	6.18	2.74	<0.001
Family history of ovarian cancer (%)	0.10	0.28	0.289
Family history of breast cancer (%)	1.99	1.98	0.927
Hormone replacement therapy use (%)	9.27	4.04	<0.001
Type of menopause (%)			<0.001
Natural	73.08	91.24	
Surgical	16.65	8.22	
Other	10.17	0.50	
Unknown	0.10	0.04	

Abbreviations: POF, premature ovarian failure; SD, standard deviation.

a Adjustment for age in ANOVA test for continuous variables and logistic regression for categorical variables.

**Wilcoxon score test.

**Table 2:** Adjusted ORs and 95%CIs for POF in the Shanghai Women's Health Study by anthropometric measurement quartiles.

POF	Quartiles of anthropometric measurements				*P*-trend
	BMI(kg/m^2^)				
	<22.2	22.2–24.43	24.44–26.84	≥26.85	
Number of cases	286	236	228	253	
Age-adjusted odds ratio	1.00	0.85(0.71,1.01)	0.82(0.69,0.98)	0.95(0.80,1.13)	0.529
Multivariate-adjusted odds ratio^a^	1.00	0.86(0.72,1.03)	0.80(0.66,0.95)	0.89(0.75,1.07)	0.171
	**WHR**				
	<0.79	0.79–0.81	0.82–0.85	≥0.86	
Number of cases	220	186	288	309	
Age-adjusted odds ratio	1.00	1.01(0.83,1.23)	1.21(1.01,1.44)	1.41(1.18,1.68)	<0.001
Multivariate-adjusted odds ratio^a^	1.00	0.98(0.80,1.20)	1.17(0.97,1.40)	1.26(1.04,1.51)	0.005

Abbreviations: POF, premature ovarian failure; OR, odds ratio; CI, confidence interval.

a Adjusted for age at study enrollment, occupation, income, current smoking (yes/no), nulliparity, age at menarche, type of menopause, and hormone replacement therapy (yes/no).

During a median follow-up of 11.2 years, 133 POF cases died (37 from CVD, 58 from cancer, and 38 from other causes), while 3,298 non-cases died (1,032 from CVD, 1,401 from cancer, and 865 from other causes) (Table 3). POF was associated with an increased risk of all-cause and cancer mortality (multivariable-adjusted HRs (95%CI): 1.29 (1.08–1.54) and 1.38 (1.05–1.81), respectively). All-cause and cancer mortality were similarly higher among women with POF who did not report using HRT. Mortality due to CVD was non-significantly increased in women who reported POF.

**Table 3:** Adjusted HRs and 95%CIs for all-cause and cause-specific mortality in POF cases.

Cause of death	Non-cases (*N* = 33,399)	POF cases	
		Overall (*N* = 1,003)	Non-users of HRT (*N* = 910)
All causes			
Number of deaths	3,298	133	130
Age-adjusted hazard ratio	1.00	1.43(1.20,1.70)	1.45(1.21,1.72)
Multivariate-adjusted hazard ratio^a^	1.00	1.29(1.08,1.54)	1.28(1.07,1.54)
Multivariate-adjusted hazard ratio^b^	1.00	1.28(1.07,1.53)	1.28(1.07,1.53)
Cardiovascular disease			
Number of deaths	1,032	37	36
Age-adjusted hazard ratio	1.00	1.23(0.88,1.71)	1.23(0.88,1.71)
Multivariate-adjusted hazard ratio^a^	1.00	1.11(0.79,1.55)	1.09(0.78,1.53)
Multivariate-adjusted hazard ratio^b^	1.00	1.10(0.79,1.55)	1.09(0.77,1.53)
Cancer			
Number of deaths	1,401	58	56
Age-adjusted hazard ratio	1.00	1.51(1.16,1.96)	1.52(1.17,1.99)
Multivariate-adjusted hazard ratio^a^	1.00	1.38(1.05,1.81)	1.37(1.04,1.80)
Multivariate-adjusted hazard ratio^b^	1.00	1.37(1.04,1.80)	1.36(1.03,1.80)

Abbreviations: POF, premature ovarian failure; HR, hazard ratio; CI, confidence interval; HRT hormone replacement therapy.

a Adjusted for age at study enrollment, occupation, income, current smoking (yes/no),age at menarche, type of menopause, nulliparity, and hormone replacement therapy (yes/no).

b Additionally adjusted for WHR.

Table 4 presents age- and multivariable- adjusted ORs for chronic disease-specific morbidity among women with POF. We observed no significant associations between POF and CVD, CHD, stroke, hypertension, or hyperlipidemia. Similarly, the prevalence of ovarian cysts, fatty liver disease, and bone fractures did not differ between women with and without POF. POF was significantly associated with an increased prevalence of type 2 diabetes mellitus and incidence of ovarian and uterine cancers (adjusted ORs (95%CI): 1.20 (1.01–1.42) and 1.89 (1.19–3.01), respectively) when adjusting only for age. These associations, however, lost statistical significance after adjustment for additional potential confounding factors. Interestingly, women with POF had a significantly increased prevalence of autoimmune diseases, including systemic lupus erythematosus and rheumatoid arthritis (age-adjusted OR (95%CI): 2.00 (1.37–2.92)) and multivariate-adjusted OR (95%CI): 1.56 (1.04–2.35)). On the other hand, POF was inversely associated with the incidence of breast cancer (multivariable-adjusted OR (95%CI): 0.59 (0.38–0.91). For all chronic disease categories, similar associations were found for all POF cases and non–HRT use POF cases with a possible exception for overall cancer incidence, in which significantly reduced risk was only found for POF cases who did not use HRT.

**Table 4:** Adjusted ORs and 95%CIs for chronic disease morbidity in women with POF in the Shanghai Women's Health Study.

Type of Disease	Non-cases (*N* = 33,399)	POF cases	
		Overall (*N* = 1,003)	Non-users of HRT (*N* = 910)
Cardiovascular disease			
Number of cases	8,154	215	201
Age-adjusted odds ratio	1.00	0.95(0.81,1.12)	0.94(0.80,1.11)
Multivariate-adjusted odds ratio^a^	1.00	0.97(0.82,1.14)	0.96(0.81,1.13)
Multivariate-adjusted odds ratio^b^	1.00	0.97(0.82,1.14)	0.96(0.81,1.13)
Coronary heart disease			
Number of cases	4,746	128	120
Age-adjusted odds ratio	1.00	0.99(0.82,1.20)	0.97(0.79,1.18)
Multivariate-adjusted odds ratio^a^	1.00	1.02(0.84,1.24)	1.02(0.83,1.25)
Multivariate-adjusted odds ratio^b^	1.00	1.01(0.83,1.23)	1.02(0.83,1.24)
Stroke			
Number of cases	3,336	97	94
Age-adjusted odds ratio	1.00	1.04(0.84,1.30)	1.05(0.84,1.31)
Multivariate-adjusted odds ratio^a^	1.00	1.01(0.81,1.26)	1.01(0.81,1.27)
Multivariate-adjusted odds ratio^b^	1.00	1.01(0.81,1.25)	1.01(0.80,1.26)
Hypertension			
Number of cases	21,987	525	488
Age-adjusted odds ratio	1.00	0.88(0.77,1.02)	0.88(0.76,1.01)
Multivariate-adjusted odds ratio^a^	1.00	0.84(0.73,0.97)	0.83(0.72,0.97)
Multivariate-adjusted odds ratio^b^	1.00	0.84(0.73,0.97)	0.83(0.72,0.97)
Hyperlipidemia			
Number of cases	6,643	165	147
Age-adjusted odds ratio	1.00	0.86(0.72,1.01)	0.84(0.70,1.00)
Multivariate-adjusted odds ratio^a^	1.00	0.86(0.73,0.1.03)	0.89(0.74,1.07)
Multivariate-adjusted odds ratio^b^	1.00	0.85(0.72,1.02)	0.88(0.73,1.06)
Diabetes mellitus			
Number of cases	5,330	170	162
Age-adjusted odds ratio	1.00	1.20(1.01,1.42)	1.22(1.02,1.45)
Multivariate-adjusted odds ratio^a^	1.00	1.11(0.93,1.32)	1.12(0.94,1.33)
Multivariate-adjusted odds ratio^b^	1.00	1.09(0.92,1.30)	1.11(0.92,1.32)
Autoimmune diseases^c^			
Number of cases	531	29	27
Age-adjusted odds ratio	1.00	2.00(1.37,2.92)	2.02(1.37,3.00)
Multivariate-adjusted odds ratio^a^	1.00	1.58(1.05,2.38)	1.55(1.02,2.37)
Multivariate-adjusted odds ratio^b^	1.00	1.58(1.04,2.38)	1.55(1.02,2.37)
Ovarian cysts			
Number of cases	1,372	39	30
Age-adjusted odds ratio	1.00	0.84(0.60,1.17)	0.76(0.52,1.10)
Multivariate-adjusted odds ratio^a^	1.00	0.83(0.58,1.18)	0.92(0.63,1.36)
Multivariate-adjusted odds ratio^b^	1.00	0.83(0.58,1.18)	0.92(0.63,1.36)
All cancers			
Number of cases	1,155	57	46
Age-adjusted odds ratio	1.00	1.81(1.37,2.38)	1.58(1.17,2.14)
Multivariate-adjusted odds ratio^a^	1.00	0.82(0.59,1.13)	0.70(0.49,0.99)
Multivariate-adjusted odds ratio^b^	1.00	0.82(0.59,1.13)	0.70(0.49,0.99)
Breast cancer			
Number of cases	851	28	27
Age-adjusted odds ratio	1.00	1.16(0.79,1.69)	1.24(0.84,1.83)
Multivariate-adjusted odds ratio^a^	1.00	0.59(0.39,0.91)	0.63(0.41,0.98)
Multivariate-adjusted odds ratio^b^	1.00	0.59(0.38,0.91)	0.63(0.41,0.98)
Ovarian and uterine cancers			
Number of cases	361	19	14
Age-adjusted odds ratio	1.00	1.89(1.19,3.01)	1.52(0.89,2.60)
Multivariate-adjusted odds ratio^a^	1.00	1.21(0.74,1.99)	1.03(0.59,1.82)
Multivariate-adjusted odds ratio^b^	1.00	1.21(0.74,1.98)	1.03(0.58,1.81)
Fatty liver			
Number of cases	5,070	136	120
Age-adjusted odds ratio	1.00	0.89(0.74,1.07)	0.89(0.73,1.08)
Multivariate-adjusted odds ratio^a^	1.00	0.88(0.72,1.06)	0.93(0.76,1.14)
Multivariate-adjusted odds ratio^b^	1.00	0.86(0.71,1.04)	0.91(0.74,1.12)
Bone fracture			
Number of cases	4,477	123	111
Age-adjusted odds ratio	1.00	0.99(0.82,1.20)	0.97(0.79,1.19)
Multivariate-adjusted odds ratio^b^	1.00	1.00(0.82,1.21)	0.99(0.80,1.21)
Multivariate-adjusted odds ratio^a^	1.00	1.00(0.82,1.21)	0.99(0.80,1.21)

Abbreviations: POF, premature ovarian failure; OR, odds ratio; CI, confidence interval.

a Adjusted for age at study enrollment, occupation, income, current smoking (yes/no),age at menarche, type of menopause, nulliparity, and hormone replacement therapy (yes/no).

b Additionally adjusted for WHR.

c Including systemic lupus erythematosus and rheumatoid arthritis.

## Discussion

In this large study of middle-aged and elderly Chinese women, 2.8% of postmenopausal women experienced menopause before 40 years of age, a lower proportion than that reported among Western women (3.7%) [22]. Furthermore, POF, which was correlated with high WHR, was found to be associated with increased risk of all-cause and cancer mortality and with a higher prevalence of autoimmune disease in this population. Not unexpectedly, breast cancer risk was reduced among women with POF. Overall cancer incidence was also reduced among POF cases who did not use HRT.

Our finding of an association of increased total mortality with POF is concordant with most studies conducted among Western populations [5], [22]–[24]. Jacobsen *et al.* reported increased all-cause mortality among women with POF in a cohort of 6,182 California Seventh-Day Adventists, compared with women whose age at menopause was 52–55 years, (adjusted HR (95%CI): 1.5 (1.0–2.3)) [5]. In another study conducted among 12,134 Dutch women, women whose menopause occurred before age 40 years had significantly increased all-cause mortality (HR (95%CI): 1.40 (1.15–1.17)) after adjustment for confounding factors, as compared with the reference group whose menopause occurred at age 50–54 years [22]. Our study, as well as the literature published to date, therefore supports an association between early menopause and increased overall mortality [25] and suggests that POF is indeed a manifestation of premature aging. Ovarian aging, as reflected by the age at which spontaneous menopause occurs, may be expected to correlate highly with the aging of other tissues and to degenerative diseases and mortality that occur as a consequence of natural aging. However, the mechanism for early ovarian aging in some women remains unclear, and we did not observe a higher frequency of other degenerative diseases such as hypertension in our study population [23]. Consistent with our observations of increased cancer mortality among women with POF, Cooper *et al.* observed that women whose age at menopause was 40–44 years experienced higher cancer mortality than women whose age at menopause was after age 50 years [24].

The relationship between early menopause and CVD or CHD in previous studies is inconsistent. Early menopause has been observed to increase mortality from CHD among 2,873 women from the Framingham cohort; women who experienced menopause before age 40 years were shown to be 53% more likely to have CHD, compared with women whose menopause occurred after age 55 years [26], [27]. Another study reported that women with menopause at age <40 years had no increase in mortality due to CHD, compared with women who experienced later menopause (at age 50–54 years) [24]. In this study, we found no statistically significant association of POF with either mortality or morbidity related to CVD.

Alterations in the immune system may induce POF secondary to the deletion of follicles or a disruption of normal ovarian function [28]. It is estimated that 20% of patients with POF have associated autoimmune disease, most commonly type I diabetes mellitus, systemic lupus erythematosus (SLE), and rheumatoid arthritis [8], [29]–[31]. Although there was no significant association between POF and diabetes after adjusting for other factors in our study, we found that POF cases were more prevalent among women with autoimmune diseases, including SLE and rheumatoid arthritis.

In a Dutch population-based breast cancer-screening cohort of 10,591 women, early menopause (occurring at age 45–49 or age 44 or younger) had a protective effect on the risk of breast cancer (HRs: 0.67 and 0.66, respectively) as compared with menopause occurring at age 55 or older [32]. Likewise, each year of delay in age at menopause was found to be associated with a 2.8% increased risk of breast cancer in a collaborative reanalysis of data from 51 epidemiological studies [33]. Consistent with these findings, we found that POF was associated with decreased risk of breast cancer. It has been suggested that this association is due to the cessation of cyclical ovarian estrogen production at menopause [33]. The cumulative life-time exposure of women with POF to endogenous circulating sex hormones is shorter than that of women with later menopause, leading to reduced risk of breast cancer over a woman's lifetime.

We found that higher WHR, a primary marker of central adiposity, was positively associated with prevalence of POF in our study. This finding is consistent with a report by Poehlman *et al.* that showed that women who experienced menopause had a greater increase in WHR (0.04±0.01) than women who remained premenopausal (0.01±0.01) after six years follow-up [34]. The principal estrogen formed in postmenopausal women, estrone, is formed by peripheral aromatization of plasma androstenedione, which is secreted by the ovaries and/or adrenal glands [35]–[37]. One of the sites of this conversion is adipose tissue, and the conversion of plasma androstenedione to estrone is positively associated with body weight [38], [39]. Higher WHR helps to maintain estrone concentrations in postmenopausal women. On the other hand, estrogen may promote the accumulation of gluteo-femoral fat (32). Low estrogen levels among women with POF may affect their body fat distribution. In our study, BMI was unrelated to POF and adjustment for WHR did not materially change the association of POF with mortality and morbidity of several major chronic diseases, indicating high WHR may be the consequence but rather the cause of POF. Nevertheless, because WHR was measured after the occurrence of POF in the current study and no information for WHR prior to POF was available, the nature of this association needs further evaluation.

Our study has several significant strengths: the study was population-based, the participation rate and follow-up rates were high, and follow-up surveys were conducted biennially to ascertain outcomes and changes in chronic disease morbidity. We were also able to adjust for a wide range of potential confounders. We excluded women with hysterectomy or hysterectomy with unilateral ovariectomy from the current analysis because not all women with these surgeries have lower estrogen levels than other women with POF [40], [41]. However, some limitations of this study must be considered. Since POF was identified based on self-reported age at menopause, it may therefore be subject to recall bias. Gonadotropin levels (*e.g.*, levels of follicle-stimulating hormone) were not measured in this study to confirm POF. Therefore, we cannot exclude the possibility that some POF is amenorrhea caused by diseases or conditions such as hypothalamic/pituitary disease, excessive exercise or dieting, androgen excess, and insulin resistance. Although, excessive exercise or dieting is rare in our study population. The relatively small sample size available for the stratified analyses also prevented us from estimating the association of POF with mortality for specific cancers and major chronic diseases other than breast and gynecologic cancers, CHD, and stroke. In addition, few women with POF used HRT, preventing an evaluation of potential effect modification by estrogen replacement. Furthermore, because anthropometrics were taken at baseline after POF occurrence, and there is a lack of information on BMI/WHR prior to POF, we can't evaluate the time sequence of the BMI/WHR and POF association. Last, information on cause of death, and diagnosis of chronic diseases (except cancer) was not verified and thus, subject to misclassification, which may bias our result towards null.

In summary, this study of urban Chinese women suggests that, while POF confers a lower risk of breast cancer, it is associated with excess all-cause and cancer mortality and with excess morbidity, including an increased likelihood of some autoimmune diseases. Women with POF may benefit from greater attention to their unique health needs, including increased cancer and chronic disease surveillance. Research is needed on whether Chinese women with POF would benefit from HRT.
